# Mobile Health Usage, Preferences, Barriers, and eHealth Literacy in Rheumatology: Patient Survey Study

**DOI:** 10.2196/19661

**Published:** 2020-08-12

**Authors:** Johannes Knitza, David Simon, Antonia Lambrecht, Christina Raab, Koray Tascilar, Melanie Hagen, Arnd Kleyer, Sara Bayat, Adrian Derungs, Oliver Amft, Georg Schett, Axel J Hueber

**Affiliations:** 1 Department of Internal Medicine 3 – Rheumatology and Immunology Friedrich-Alexander University Erlangen-Nürnberg Erlangen Germany; 2 Chair of Digital Health Friedrich-Alexander University Erlangen-Nürnberg Erlangen Germany; 3 Section Rheumatology Sozialstiftung Bamberg Bamberg Germany

**Keywords:** mobile applications, eHealth, rheumatology, mHealth, eHEALS, telemedicine

## Abstract

**Background:**

Mobile health (mHealth) defines the support and practice of health care using mobile devices and promises to improve the current treatment situation of patients with chronic diseases. Little is known about mHealth usage and digital preferences of patients with chronic rheumatic diseases.

**Objective:**

The aim of the study was to explore mHealth usage, preferences, barriers, and eHealth literacy reported by German patients with rheumatic diseases.

**Methods:**

Between December 2018 and January 2019, patients (recruited consecutively) with rheumatoid arthritis, psoriatic arthritis, and axial spondyloarthritis were asked to complete a paper-based survey. The survey included questions on sociodemographics, health characteristics, mHealth usage, eHealth literacy using eHealth Literacy Scale (eHEALS), and communication and information preferences.

**Results:**

Of the patients (N=193) who completed the survey, 176 patients (91.2%) regularly used a smartphone, and 89 patients (46.1%) regularly used social media. Patients (132/193, 68.4%) believed that using medical apps could be beneficial for their own health. Out of 193 patients, only 8 (4.1%) were currently using medical apps, and only 22 patients (11.4%) stated that they knew useful rheumatology websites/mobile apps. Nearly all patients (188/193, 97.4%) would agree to share their mobile app data for research purposes. Out of 193 patients, 129 (66.8%) would regularly enter data using an app, and 146 patients (75.6%) would welcome official mobile app recommendations from the national rheumatology society. The preferred duration for data entry was not more than 15 minutes (110/193, 57.0%), and the preferred frequency was weekly (59/193, 30.6%). Medication information was the most desired app feature (150/193, 77.7%). Internet was the most frequently utilized source of information (144/193, 74.6%). The mean eHealth literacy was low (26.3/40) and was positively correlated with younger age, app use, belief in benefit of using medical apps, and current internet use to obtain health information.

**Conclusions:**

Patients with rheumatic diseases are very eager to use mHealth technologies to better understand their chronic diseases. This open-mindedness is counterbalanced by low mHealth usage and competency. Personalized mHealth solutions and clear implementation recommendations are needed to realize the full potential of mHealth in rheumatology.

## Introduction

Rheumatoid arthritis, psoriatic arthritis, and axial spondyloarthritis are complex pathogenetic chronic diseases. These disease entities also require a complex treatment structure with interdisciplinary care by various specialists (rheumatologists, dermatologists, gastroenterologists, ophthalmologists, physiotherapists, etc), and intensive and regular disease monitoring is essential. With this complexity, affected patients often report a lack of understanding of their disease [1].

Mobile health (mHealth) holds promise to improve health care delivery and outcomes for people with chronic diseases [2]. Ideally, mHealth solutions, such as mobile apps and wearable sensors could empower patients, provide individual support, and lead to better outcomes than those available through standard care. Patients with chronic rheumatic diseases already have access to a broad range of mHealth solutions, starting from symptom checkers [3] or referral tools [4]. Once a diagnosis is established, mHealth tools enable patients to better monitor their symptoms passively through sensors [5] and actively by entering data [6,7]. Furthermore, electronic medication reminders can increase medication adherence [8,9], and supporting digital therapy can reduce pain [9] and improve important comorbidities (eg, depression) [10].

Recently the European League Against Rheumatism and Working Group Young Rheumatology of the German Society for Rheumatology (*Arbeitsgemeinschaft Junge Rheumatologie der Deutschen Gesellschaft für Rheumatologie*) published recommendations for the development of mobile apps in rheumatology [11,12]. The early integration of patients in the app development process was stressed in both papers. However, little is known about the patient perspective on mHealth solutions, as current literature focuses on rheumatologists [11,13,14]. To successfully integrate the various mHealth solutions into clinical routine, it is essential to identify barriers and the needs of patients.

The aim of this study was to explore mHealth usage, preferences, barriers, and eHealth literacy reported by German patients living with rheumatic diseases.

## Methods

Between December 2018 and January 2019, consecutive patients seen at one rheumatology outpatient clinic of the University Hospital Erlangen were asked to complete a paper-based survey. This study was approved by the Ethics committee (No. 418-18B) and conducted referring to good clinical practice. All patients provided informed consent.

To create the survey (Multimedia Appendix 1), a broad literature review was carried out. Previous mHealth patient surveys in oncology [15] and rheumatology [16] served as a starting basis. The survey comprised four main parts: (1) sociodemographics and health characteristics, (2) mHealth preferences and usage, (3) eHealth literacy, and (4) communication and information preferences. The survey was pilot-tested with 10 patients to detect necessary formatting and wording changes. Minor revisions were made accordingly. Foreign words and technical term explanations were provided in a footnote. Inclusion criteria were patients (1) aged ≥18 years, (2) who were literate in German, (3) who had the physical and mental ability to fill out a structured questionnaire, and (4) who fulfilled classification criteria for rheumatoid arthritis [17], axial spondyloarthritis [18], or psoriatic arthritis [19].

The sociodemographic and health characteristics included age; gender; residence; diagnosis; disease duration; patient global assessment of disease activity; and current usage of smartphones, tablets, activity trackers, and social media.

The mHealth preferences and usage section included questions about the preferred time and frequency for using a rheumatology app. Patients were asked to rate their preference of app features (5-point Likert scale) and rate the importance of app characteristics (10-point Likert scale). Inquiries were made on internet usage and perceived usefulness, willingness to share recorded app data, general perception about the utility of medical apps, web-based services for improving patient’s health, and telemedicine.

Patients’ eHealth literacy was measured using the validated German version [20] of the eHealth Literacy Scale (eHEALS) [21]; eHEALS has been translated and validated in multiple languages [20-22]. It is based on a 5-point Likert scale and includes 8 statements concerning self-perceived eHealth literacy.

Patients were asked to state their preferences concerning medication reminders, medical information format, digitally provided information structure, patient diary type, and physician communication type. Rankings did not have to be unique. Characteristics were summarized using means, standard deviations, counts, and percentages as appropriate. We used Pearson correlation to explore relationships between continuous variables. The relationship between the eHEALS score and internet use frequency was examined using a linear regression model with the eHEALS score as the dependent variable and internet use as an ordinal predictor encoded using orthogonal polynomials to characterize nonlinear effects. Relationships between eHEALS score and binary preferences were examined using logistic regression. All models included age and gender adjustments. Two-sided *P* values less than .05 were considered significant. We used Excel (Microsoft Corp) and R (version 3.5.3; R Foundation for Statistical Computing) for data manipulation and analyses.

## Results

### Patient Characteristics

In total, 224 patients were recruited. Only complete surveys (N=193) were considered for the final analysis. The number of patients rejecting participation was not measured. The study sample’s demographics are shown in Table 1. Mean age was 52.1 (SD 13.7) years, with 34.7% (67/193) being at least 60 years old; 59.6% (115/193) were female, and 53.9% (104/193) had been diagnosed with rheumatoid arthritis. The mean disease duration was 8.3 years (SD 8.0). Nearly all patients regularly used a smartphone (176/193, 91.2%), and nearly half of the patients regularly used a tablet (86/193, 44.6%) and social media (89/193, 46.1%). Only a minority used activity trackers (20/193, 10.4%).

**Table 1:** Demographic and health characteristics.

Characteristic		Values (N=193)	
Age (years), mean (SD)		52.1 (13.7)	
**Age (years), n (%)**			
	18-39		46 (23.8)
	40-59		80 (41.5)
	≥60		67 (34.7)
**Gender n (%)**			
	Female		115 (59.6)
	Male		78 (40.4)
**Diagnosis, n (%)**			
	Rheumatoid arthritis		104 (53.9)
	Axial spondyloarthritis		37 (19.2)
	Psoriatic arthritis		52 (26.9)
Patient global assessment of disease activity (0-10), mean (SD)		3.8 (2.4)	
Disease duration (years), mean (SD)		8.3 (8.0)	
**Disease duration (years), n (%)**			
	≤1		42 (21.8)
	2-5		44 (22.8)
	>5		107 (55.4)
**Residence, n (%)**			
	Village		74 (38.3)
	Small city		48 (24.9)
	Midsized city		35 (18.1)
	Big city		36 (18.7)
**Regular usage, n (%)**			
	Smartphone		176 (91.2)
	Tablet		86 (44.6)
	Activity tracker		20 (10.4)
	Social media		89 (46.1)
	Medical apps		8 (4.1)

### Medical App Acceptance and Willingness to Provide mHealth Data for Research Purposes

**Figure 1:**
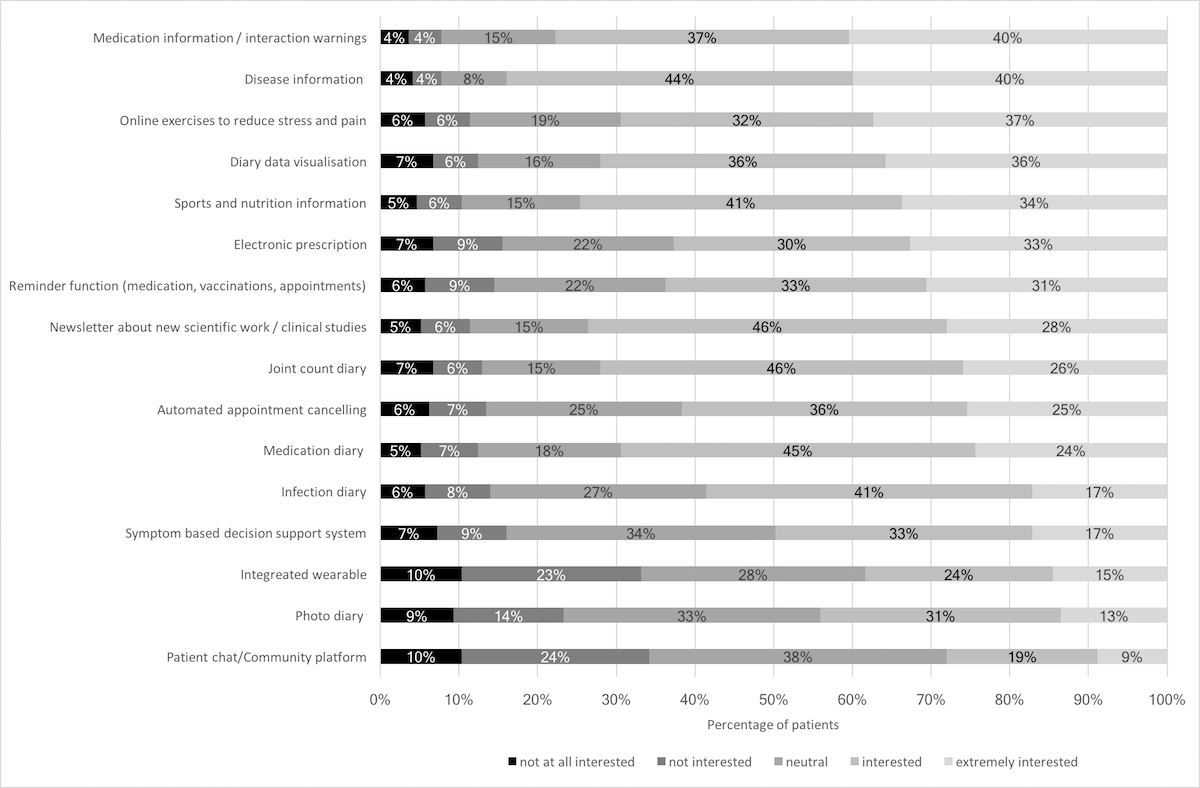
App function preferences (responses to "What app functions would you like?").

Preferences and attitudes regarding potential mHealth apps and data flow were addressed (Table 2). More than two-thirds of the patients (132/193, 68.4%) believed that medical apps are helpful for their health; however, only 4.1% (8/193) patients currently used medical apps, of which none were rheumatology specific apps. Increasing eHEALS scores were associated with a higher probability of expressing belief that apps were helpful after adjusting for age and gender (odds ratio [OR] 1.13, 95% CI 1.07 to 1.19, *P*<.001). Nearly all patients (188/193, 97.4%) were willing to transfer app data for research purposes, if data security would be ensured. The main barrier for sharing app data with the physician was that personal contact was considered as sufficient (53/193, 63.9%). Second, patients (42/193, 50.6%) were concerned about data transfer. The majority of patients (174/193, 90.2%) wanted to be contacted in case an app detected an abnormality concerning their health, whereas 57.0% (110/193) were also willing to transfer app data to the treating physician. Concerns were data usage, storage, and transfer. Only 28.0% (54/193) were interested in comparing their medication adherence to the medication adherence of other patients. Regarding official app recommendations, 75.6% (146/193) of interviewed patients wanted advice from the national society of rheumatology. Weekly data entry was preferred by 30.6% (59/193) with durations of 5-15 minutes (65/193, 33.7%). Measured on a scale of 10, the most important app characteristics were security (mean 8.9, SD 2.5) and usability (mean 8.5, SD 2.5) (Table 2). Regarding preferred app functions, patients were most interested in information about medications and diseases and were least interested in direct exchange such as chats with peers with the same disease (Figure 1).

**Table 2:** Patient attitudes towards medical apps.

Characteristic		Values (N=193)
Patients believing medical apps are helpful for their health, n (%)		132 (68.4)
Patients willing to transfer app data for research purposes, n (%)		188 (97.4)
Patients willing to transfer data to physician with app, n (%)		110 (57.0)
**Reason for not willing to transfer data to physician with app, n (%)^a^**		
	I don't have a suitable device	13 (15.7)
	I don't have the technical skills	23 (27.7)
	I have concerns about data usage	33 (39.8)
	I have concerns about data storage	31 (37.3)
	I have concerns about data transfer	42 (50.6)
	I have concerns about data protection	24 (28.9)
	I only want personal contact with physician	53 (63.9)
	I don't find this useful	11 (13.3)
Patients who want to be contacted in case of app-monitoring abnormalities		174 (90.2)
Patients interested to compare medication adherence to other patients, n (%)		54 (28.0)
Patients who want official app recommendations from national society of rheumatology, n (%)		146 (75.6)
**Preferred frequency of app usage, n (%)**		
	Not at all	64 (33.2)
	Daily	11 (5.7)
	Weekly	59 (30.6)
	Monthly	37 (19.2)
	Each 3 months	18 (9.3)
	Less frequently	4 (2.1)
**Preferred time of app usage (minutes), n (%)**		
	Not at all	64 (33.2)
	0-5	45 (23.3)
	5-15	65 (33.7)
	15-30	18 (9.3)
	>30	1 (0.5)
**Importance of app characteristics (0-10), mean (SD)**		
	Interactivity	4.5 (2.9)
	Design	4.7 (3.2)
	Usability	8.5 (2.5)
	Data security	8.9 (2.5)

^a^Multiple answers were possible.

### Internet Usage and Perceived Usefulness

To address habits regarding information search, patients were questioned concerning internet usage. The majority of patients (168/193, 87.0%) had previously used the internet to obtain health information (Table 3), and the remaining patients (25/193, 13.0%) lacked the skills or motivation to do a search; 7/25 (28.0%) had no internet access, 7/25 (28.0%) did not think it would be helpful, 6/25 (24.0%) found the information from their physician sufficient, 2/25 (8.0%) did not know how to do a search, and 3/25 (12.0%) stated another reason. Some patients had previously communicated with a physician by email (56/193, 29.0%). Participation in an online health program was rare (3/193, 1.6%). Online support groups were used by patients to post information (14/193, 7.3%), chat with other patients (19/193, 9.8%), or read information (85/193, 44.0%); 19.7% (38/193) were aware of the medication website of the German Society of Rheumatology (*Deutsche Gesellschaft für Rheumatologie*).

**Figure 2:**
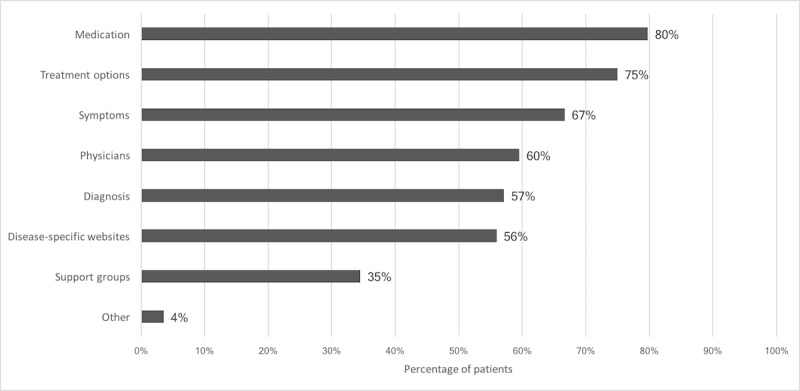
Information previously searched on the internet (responses to "What health information did you look for on the internet?).

Figure 2 presents the type of health information searched on the internet. Most patients looked for medication information (134/168, 79.8%) whereas information on support groups was the least commonly searched (58/168, 34.5%); 124/193 (64.2%) patients preferred filling out medical questionnaires electronically before their clinical visits, 102/193 (52.8%) patients preferred receiving a doctor’s letter in an electronic format instead of paper, and 98/193 (50.2%) patients preferred communicating with their rheumatologist by a video call.

**Table 3:** Internet usage, perceived usefulness, and eHealth literacy.

Characteristic		Values (N=193)
**Did you previously look for health information on the internet? n (%)**		
	Yes	168 (87.0)
	No	25 (13.0)
**Did you previously communicate with a physician by email? n (%)**		
	Yes	56 (29.0)
	No	137 (71.0)
**Did you previously participate in an online health program? n (%)**		
	Yes	3 (1.6)
	No	190 (98.4)
**Did you use an online supporting group before to...?^a^** **n (%)**		
	Post information	14 (7.3)
	Chat with patients	19 (9.8)
	Read information	85 (44.0)
	I never used one before	103 (53.4)
**Do you know useful rheumatology websites or apps? n (%)**		
	Yes	22 (11.4)
	No	171 (88.6)
**Do you know the DGRh^b^** **medication information website? n (%)**		
	Yes	38 (19.7)
	No	155 (80.3)
**How useful do you find the internet to make health-related decisions? n (%)**		
	Not useful at all	17 (8.8)
	Not useful	19 (9.8)
	Unsure	74 (38.3)
	Useful	72 (37.3)
	Very useful	11 (5.7)
eHealth literacy, mean (SD)		26.3 (7.1)

^a^Multiple answers were possible.

^b^DGRh: Deutsche Gesellschaft für Rheumatologie (German Society of Rheumatology).

### eHealth Literacy

**Figure 3:**
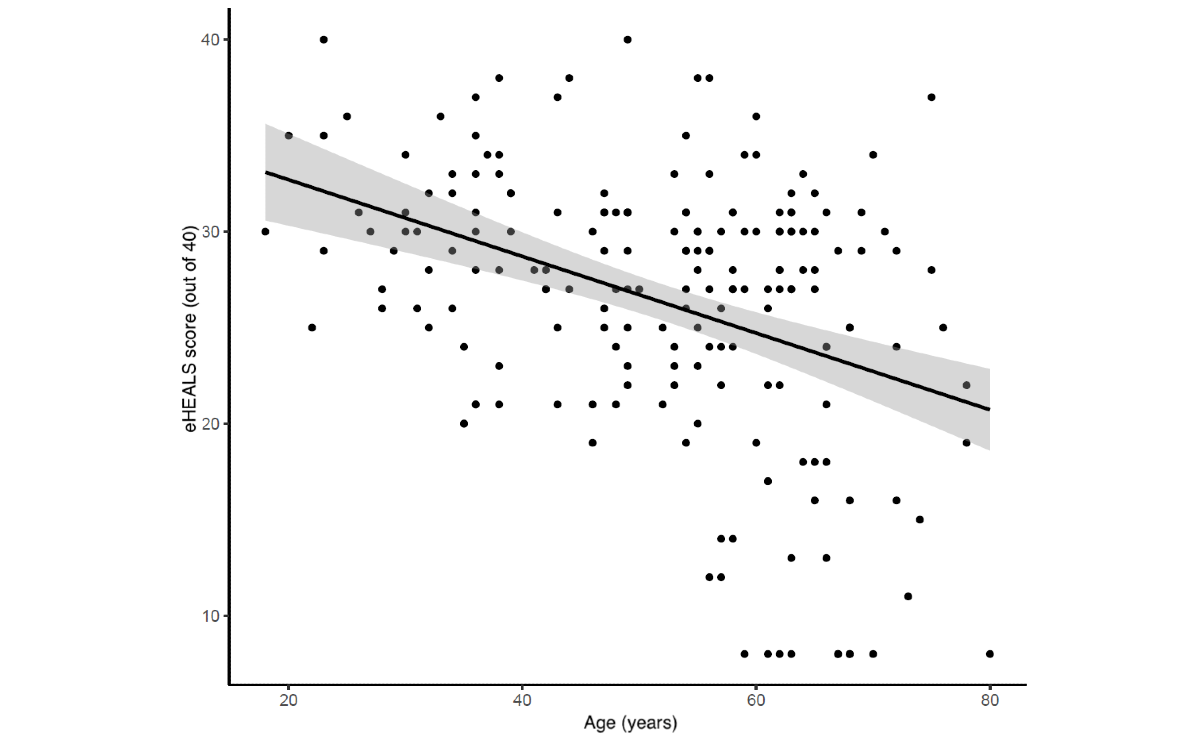
Negative relationship between eHEALS score and age.

**Figure 4:**
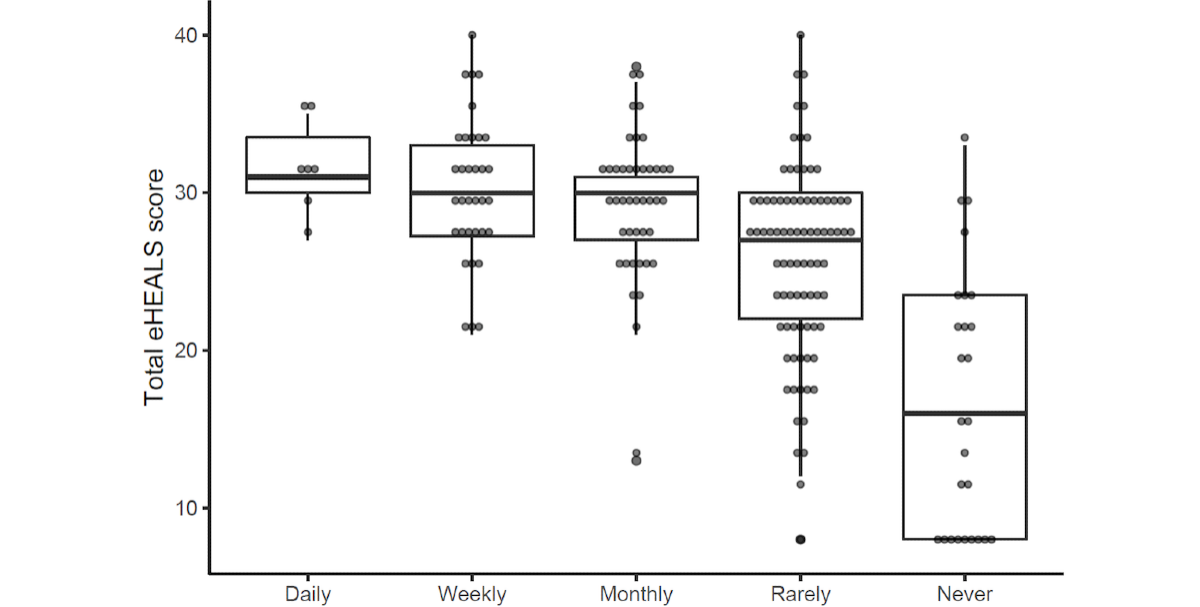
Association of eHEALS score and frequency of internet use.

**Figure 5:**
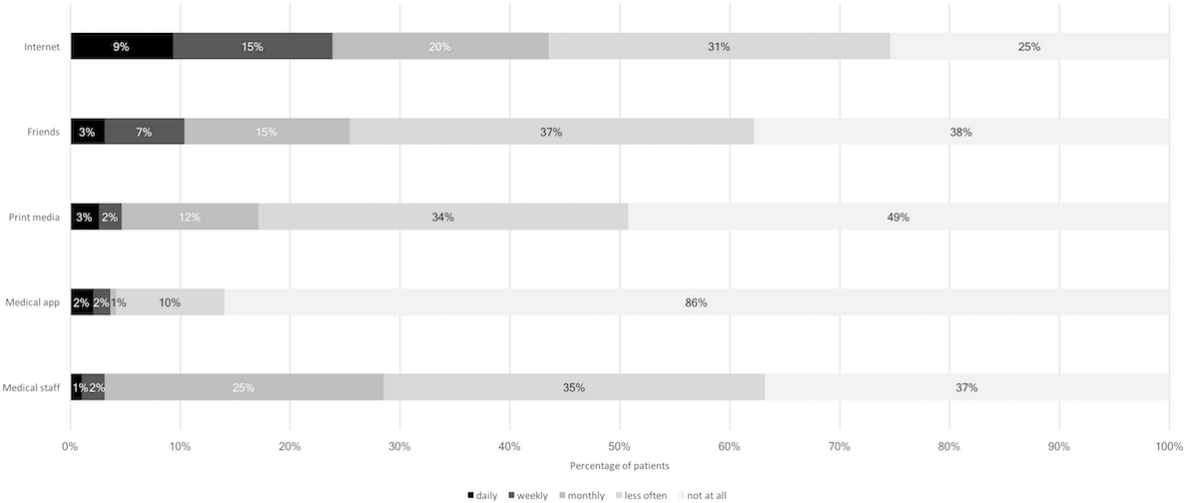
Health information sources used prior to clinical visits (responses to "How often did you use one of the following options to receive health information in the last three months?").

Mean eHealth literacy using eHEALS was 26.3 (SD 7.1) out of 40. Mean scores in women and men were 25.8 and 27.0, respectively, with a mean difference of –1.15 (95% CI –3.14 to 0.84) showing no important effect of gender. Age showed a negative correlation (r=–0.38, 95% CI –0.5 to –0.26) with the eHEALS score (Figure 3). Table 4 shows the distribution of responses to the 8 eHEALS items. The majority of the patients agreed that they know how to use the internet to answer their questions about health (82/193, 71.5%), and a considerable proportion of patients (82/193, 42.5%) felt uncomfortable using information from the internet to make health decisions. A lower eHEALS score was associated with a decreasing frequency of internet usage; the regression analysis shows that, after adjustment for age and gender, the negative association with decreasing frequency of internet use and eHEALS score followed a second-order polynomial (linear: –8.87, 95% CI –11.97 to –5.76; quadratic:–5.64, 95% CI –8.37 to –2.92) meaning that each step of decrease in the use-frequency categories was associated with, not an equal, but a progressively greater decrease in the eHEALS score (Figure 4). Figure 5 shows the usage frequency of different health information sources during the last 3 months prior to the clinical visit. The internet was the most frequently used information source (144/193, 74.6%), with 9.3% (18/193) using it daily, 14.5% (28/193) weekly, and 19.7% (38/193) monthly.

**Table 4:** eHealth literacy.

eHEALS item	Participants (N=193), n (%)			Score
	Strongly disagree or disagree	Neutral	Agree or strongly agree	Mean (SD)
I know how to find helpful health resources on the Internet	30 (15.5)	50 (25.9)	113 (58.5)	3.5 (1.1)
I know how to use the Internet to answer my questions about health	30 (15.5)	25 (13.0)	138 (71.5)	3.6 (1.1)
I know what health resources are available on the Internet	51 (26.4)	68 (35.2)	74 (38.3)	3.1 (1.1)
I know where to find helpful health resources on the Internet	47 (24.4)	57 (29.5)	89 (46.1)	3.2 (1.1)
I know how to use the health information I find on the Internet to help me	48 (24.9)	49 (25.4)	96 (49.7)	3.2 (1.1)
I have the skills I need to evaluate the health resources I find on the Internet	26 (13.5)	30 (15.5)	137 (71.0)	3.8 (1.1)
I can tell high quality health resources from low quality health resources on the internet	37 (19.2)	52 (26.9)	104 (53.9)	3.4 (1.1)
I feel confident in using information from the Internet to make health decisions	82 (42.5)	82 (42.5)	29 (15.0)	2.6 (1.0)

### Conservative Communication Preferences

The preferred way to contact the treating rheumatologist was via telephone (136/196, 69.4%), email (42/196, 21.4%), and chat (8/196, 4.1%) (Table 5, last panel). Most patients wanted to be reminded of regular medication intake (198/260, 76.2%). The preferred method for reminders was via an app (118/260, 45.4%) (Table 5). The large majority of patients (173/188, 92.0%) wanted medical information and the preferred media were paper (104/188, 55.3%), app (40/188, 21.3%), and website (29/188, 15.4%). Concerning digitally provided information, patients preferred plain text and images (160/196, 81.6%) over exchange functions (29/196, 14.8%) and game-based learning (7/196, 3.6%). The majority of patients preferred using a patient diary (167/190, 87.9%) (preference for paper was greater than that for an app, which was greater than that for a website). App usage preference increased over the last years (5.4% to 18.1% from 2014 to 2018, see Multimedia Appendix 2).

**Table 5:** Preferences for medication reminders, medical information format, digitally provided information structure, patient diary type, and physician communication type.

Characteristic^a^			Preference (N=193), n (%)				Score
			First		Last		Mean (SD)
**I would like to be reminded of the regular medication intake by...^b^**							
	Short message (SMS)	39 (15.0)		13 (6.0)		2.8 (1.4)	
	Mobile app	118 (45.4)		17 (7.9)		3.1 (1.7)	
	Email	18 (6.9)		10 (4.6)		3.2 (1.3)	
	Telephone call	20 (7.7)		28 (13.0)		3.5 (1.6)	
	Not wanted	62 (23.8)		78 (36.1)		3.8 (2.2)	
	Postcard	3 (1.2)		70 (32.4)		4.9 (1.3)	
**I prefer medical information ...^c^**							
	On paper	104 (55.3)		12 (6.2)		1.8 (1.0)	
	On a website	29 (15.4)		16 (8.2)		2.3 (0.8)	
	In an app	40 (21.3)		15 (7.7)		2.4 (0.9)	
	Not wanted	15 (8.0)		151 (77.8)		3.6 (0.9)	
**For digitally provided information (website/app) this would be important to me:^d^**							
	Text- and image-based information	160 (81.6)		16 (8.1)		1.3 (0.6)	
	Exchange with others	29 (14.8)		61 (31.0)		2.2 (0.7)	
	Game-based learning	7 (3.6)		120 (60.9)		2.6 (0.6)	
**I would document my state of health and tablet intake in a “patient diary”...^c^**							
	On paper	92 (48.4)		14 (7.2)		1.9 (1.0)	
	In an app	53 (27.9)		19 (9.7)		2.2 (0.9)	
	On a website	22 (11.6)		31 (15.9)		2.6 (0.9)	
	Not wanted	23 (12.1)		131 (67.2)		3.3 (1.1)	
**My preferred way to contact my rheumatologist is via...^c^**							
	Telephone call	136 (69.4)		8 (4.0)		1.5 (0.8)	
	Email	42 (21.4)		5 (2.5)		2.0 (0.7)	
	Website/chat	8 (4.1)		32 (16.2)		2.9 (0.7)	
	Not wanted	10 (5.1)		153 (77.3)		3.6 (0.8)	

^a^Answer options could be ranked with the same preference level.

^b^1=preferred option, 6=least preferred option.

^c^1=preferred option, 4=least preferred option.

^d^1=preferred option, 3=least preferred option.

## Discussion

### Main Findings

Patients with rheumatic and musculoskeletal diseases are ready and willing to use mHealth technologies. Patients believe in the potential of mHealth and are open to participating actively by sharing health data with their physicians and researchers. Structured electronic data acquisition holds great promise to increase data quality, increase quantity, and reduce missing data and bureaucracy. Patients preferred filling out questionnaires before clinical visits, using video calls, and receiving electronic doctor’s letters. These measures could drastically improve the clinical experience for patients, cutting down waiting time and long drives to the hospital. For clinicians, automatic data import could reduce administrative efforts. Furthermore, continuously obtained mHealth patient data increases the basis for shared personalized clinical decision making.

Some patients, however, prefer personal contact with their physician and were concerned about data storage and transfer. Also, at the time of the study, no single patient was using a rheumatology specific app, only 4.1% were using medical apps at all, and 11.4% were aware of useful rheumatology websites or apps. The currently scarce mHealth usage and low eHealth literacy highlight an important need for structured mHealth guidance and patient-adapted information and education. The majority of patients clearly stated this by calling for official app recommendations from the national society of rheumatology. The most popular app features were information about medication and rheumatic diseases. The fact that 87.0% previously searched for various health information on the internet, and that the internet is the most frequently used source for health information, further supports the need for more, better, and personalized medical information. This information should be accurate and reliable, requiring rheumatologists and societies to actively lead and supervise mHealth in rheumatology. The opportunity to support therapeutic online health programs is currently not being fully utilized, despite promising study results [23]. Furthermore, our study shows an enthusiasm on the patients’ side to improve medication adherence via mobile apps and diaries. Sharing of this mHealth data with a large research registry would represent an enormous potential to improve treatments and rheumatology research. These results may help patients, developers, clinicians, and researchers to use the full potential of mHealth in rheumatology.

### Limitations

The cross-sectional design, self-reported data, and sampling method with relatively small sample size were the most important limitations of this study. Therefore, the results might not be generalizable, and actual mHealth usage might, for example, differ compared to self-reported usage.

### Comparison With Prior Work

To our knowledge, this is the first work depicting a German mHealth patient perspective in rheumatology. This work may inform mHealth policy recommendations and adds to the growing body of eHealth rheumatology knowledge [11-14,24,25] by providing detailed patient preferences, needs, and barriers. Hence, we believe that the results of this study could help in devising mHealth solutions that can be integrated into the clinical routine of patients with rheumatic diseases. The importance of including patients in the app development process is stressed in various recommendations [11,12]. *Rheuma-Auszeit* was reported as the only app developed with major patient involvement and scored as the highest quality app [11]. It was also shown that the participation of patients in app development leads to high usage; for example, the ArthritisPower app was used by >18,000 patients with rheumatic diseases in 2018 [26].

Health literacy is critical for patient empowerment [27]. Our work confirms the lack of adequate eHealth skills in rheumatology patients [28], reflected by the borderline mean eHEALS score of 26.3 out of 40. Cutoffs for the eHEALS score show considerable variation in the literature [29-31]; however, a value of 26 was set in a previous study as the cutoff for low eHealth literacy [31]. eHealth literacy was associated with a greater belief in the usefulness of medical apps, and usage of the internet to obtain health information, similar to findings reported by Noblin et al [32]. A previous study [33] showed that younger age and higher eHealth literacy correlate with perceived effectiveness of medical apps. eHealth literacy can change over time [34], and supporting programs should be implemented to increase eHealth literacy [35]. Many mHealth challenges could be overcome if more support was provided by health providers [36].

Patients demanded clear recommendations from the national society for rheumatology and expressed the highest confidence in an app developed by a rheumatic disease scientific society [25]. Our results suggest that patient app entries should take no longer than 15 minutes and should not be requested more often than weekly. Data transfer should be clearly explained to patients to eliminate this barrier. Customizable app features are needed, as preferences differ, and a one-size-fits-all approach seems to be less effective. App building blocks (videos, information, tools) should be provided so that redundant work is reduced, and a large variety of features can be provided. Ideally, the app content should be created in a joint effort by all stakeholders.

The discrepancy between app use and general belief in usefulness and interest reflect the currently unmet need of effective mHealth solutions, guidance, and education. This discrepancy is also highlighted by the dominance of conservative communication preferences. These results should carefully be interpreted, as most patients currently do not have experience with app-based communication.

In an effort to address this need, the French society for rheumatology recently developed the free patient app Hiboot, which provides patients with trustworthy information about medication and answers to frequently asked questions [37]. App usage was very low among patients (4.1%) compared to medical app usage among German rheumatologists (49%) [13] and an international group of rheumatic patients (79/394, 20%). In contrast to rheumatologists [13], and an international group of rheumatic patients (188/394, 47%) [25]; patients were not aware of any rheumatology specific apps. These results could partly be explained by the paper-based nature of the survey that enabled us to include elderly patients that might have been reluctant to join the study otherwise.

The lack of patient interest was previously identified as a major barrier to app prescription among general practitioners [38]. Besides education and careful evaluation of current mHealth solutions [11], solid evidence for the effectiveness and usability is needed to overcome current barriers and increase app prescription rate.

Concerning app feature preferences, our results were very much in line with previous research [16,25]. Patients do not prefer patient-to-patient communication features; however, patients are very interested in other app features, particularly those providing information.

As reported in previous studies [25,28], our work clearly shows that most patients regularly use the internet to retrieve medical information. In a previous study [3] published in 2016, 47% of the patients consulted the internet to investigate their symptoms. This proportion seems to be increasing, as in our study 67% and in another recent study [25] 95% of patients stated they did so. Compared to German oncology patients interviewed in 2016 [15], more rheumatic patients seem to use mobile devices regularly (69.6% compared to 91.2%); however, the information quality is very heterogeneous which could be misleading.

The obvious enthusiasm of patients to share app data for research purposes and their wish for such data to be made available to the treating rheumatologist for routine care underlines that an improvement to the science and practice of rheumatology could be realized using mHealth. A digital approach allows the inclusion of well-structured patient-generated data to improve clinical shared decision making and clinical research [39]. The interoperability of systems is crucial for the success of such mHealth tools. A part of patients want rheumatologists to review their app input. This could easily cause information overload and an additional workload to physicians; however, rationing appointments, earlier detection, and response to flares would also be possible [40]. In combination with wearable sensors and algorithms, patient monitoring could further be improved.

The medical community, public health system, and private sector need to increase their efforts to improve eHealth competencies and to provide safe and effective digital tools to leverage the way of mHealth into routine rheumatology care.

### Conclusion

To our knowledge, this is the first study capturing a detailed mHealth perspective of patients with rheumatic diseases, which could guide rheumatology app development and implementation. Most patients included in this study possessed smartphones and believed that using medical apps could be beneficial for their health. A substantial majority was also willing to share app data for research purposes. The current usage of mHealth among rheumatic patients is, however, very limited and eHealth literacy was rather poor. We could successfully identify unmet needs and patient priorities, which can be used to accelerate and guide the way of mHealth into routine rheumatology care.

## Appendix

Multimedia Appendix 1 Original German survey.

Multimedia Appendix 2 2014 and 2018/2019 Preferences for medication reminders, medical information format, digitally provided information structure, patient diary type, and physician communication type of patients with rheumatoid arthritis.

## Abbreviations

eHEALS: eHealth Literacy Scale
mHealth: mobile health
OR: odds ratio
