# Supplementary figures and images for: Mobile Health Usage, Preferences, Barriers, and eHealth Literacy in Rheumatology: Patient Survey Study
Source: JMIR Mhealth Uhealth. 2020 Aug 12;8(8):e19661. doi: 10.2196/19661 (PMC7450373; doi:10.2196/19661)

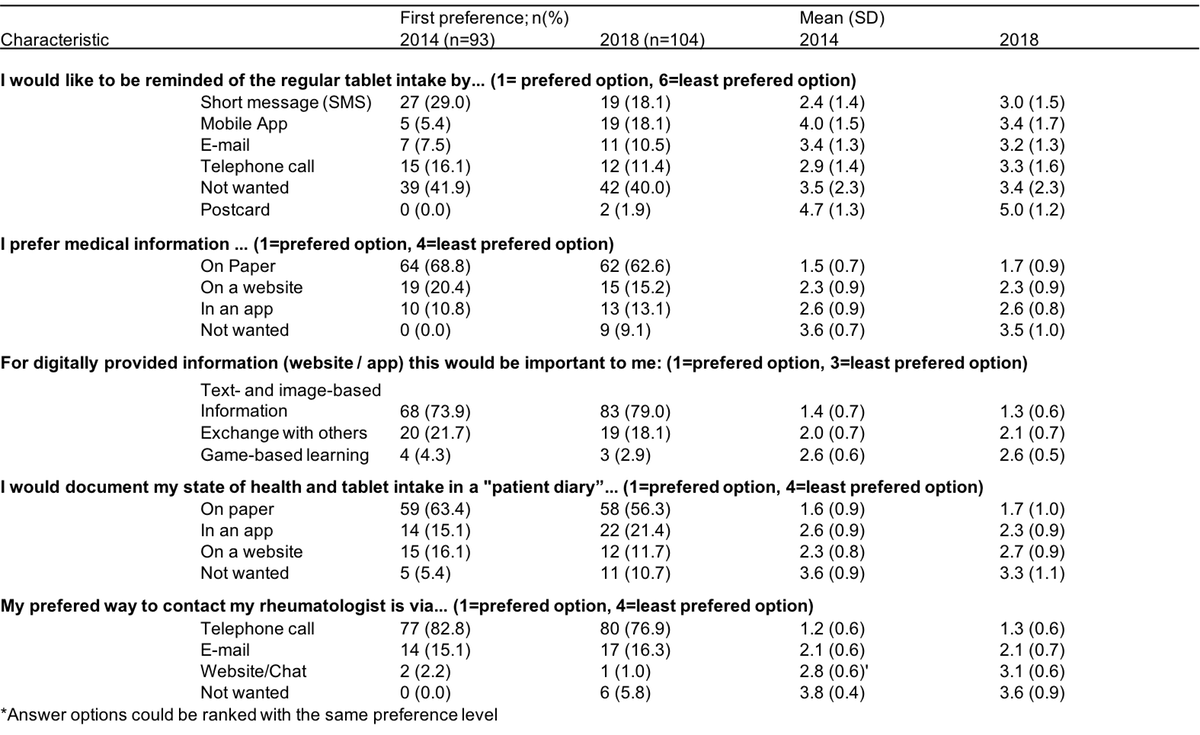

Supplement: Multimedia Appendix 2 [file mhealth_v8i8e19661_app2.png]
